# Quantitative Detection of *Borrelia burgdorferi* sensu lato in Erythema Migrans Skin Lesions Using Internally Controlled Duplex Real Time PCR

**DOI:** 10.1371/journal.pone.0063968

**Published:** 2013-05-16

**Authors:** Maria O’Rourke, Andreas Traweger, Lara Lusa, Dasa Stupica, Vera Maraspin, P. Noel Barrett, Franc Strle, Ian Livey

**Affiliations:** 1 Vaccine R&D, Baxter Bioscience, Orth/Donau, Austria; 2 Faculty of Medicine, Institute for Biostatistics and Medical Informatics, Ljubljana, Slovenia; 3 Department of Infectious Diseases, University Medical Centre Ljubljana, Ljubljana, Slovenia; 4 Paracelsus Medical University, Spinal Cord Injury and Tissue Regeneration Center Salzburg, Institute of Tendon and Bone Regeneration, Salzburg, Austria; The Johns Hopkins University School of Medicine, United States of America

## Abstract

*B. burgdorferi sensu stricto*, *B. afzelii*, *B. garinii* and *B. bavariensis* are the principal species which account for Lyme borreliosis (LB) globally. We have developed an internally controlled duplex quantitative real time PCR assay targeting the *Borrelia* 16S rRNA and the human RNAseP genes. This assay is well-suited for laboratory confirmation of suspected cases of LB and will be used to assess the efficacy of a vaccine against LB in clinical trials. The assay is highly specific, successfully detecting DNA extracted from 83 diverse *B. burgdorferi* sensu lato strains representing all major species causing LB, while 21 unrelated microbial species and human genomic DNA tested negative. The assay was highly reproducible and sensitive, with a lower limit of detection of 6 copies per PCR reaction. Together with culture, the assay was used to evaluate paired 3 mm skin biopsy samples taken from 121 patients presenting with solitary erythema migrans (EM) lesion. PCR testing identified more positive biopsy samples than culture (77.7% PCR positive versus 55.1% culture positive) and correctly identified all specimens scored as culture positive. OspA-based typing identified the majority of isolates as *B. afzelii* (96.8%) and the bacterial load was significantly higher in culture positive biopsies than in culture negative biopsies (*P*<0.001). The quantitative data also enabled relationships between *Borrelia* burden and patient symptoms to be evaluated. The bacterial load was significantly higher among patients with systemic symptoms than without (*P* = 0.02) and was significantly higher for biopsies retrieved from patients with EM lesions with central clearing (*P*<0.001). 16S copy numbers were moderately lower in samples from patients reporting a history of LB (*P* = 0.10). This is the first quantitative PCR study of human skin biopsies predominantly infected with *B. afzelii* and the first study to demonstrate a clear relationship between clinical symptoms in *B. afzelii-*infected patients and *Borrelia* burden.

## Introduction

*Borrelia burgdorferi* sensu lato (s.l.) is transmitted through the bite of infected ticks in the *Ixodes* family and is the causative agent of Lyme borreliosis (LB), the most common vector borne disease in Europe and North America. *Borrelia burgdorferi* s.l. currently comprises a clade of 17 named species, the most recent addition being *B. finlandensis sp. nov.* [1]. *B. burgdorferi* sensu stricto (s.s) is the sole species known to cause human infection in the United States, whereas in Europe *B. afzelii*, *B. garinii* (including recently designated *B. bavariensis sp. nov* [2]) and *B. burgdorferi* s.s are responsible for most cases of LB [3]. However, *B. spielmanii* [4], [5], *B. valaisiana* [6], [7], *B. bissettii* [8], [9] and *B. lusitaniae* [10], [11] have been detected in samples of human skin and cerebrospinal fluid, suggesting that these species can also give rise to LB.

Although it is not routine practice to request laboratory testing to confirm LB presenting as erythema migrans (EM) [3], laboratory testing is highly valuable when assessing vaccine efficacy in clinical trials [12]. Moreover, demonstration of *Borrelia* in tissues is also valuable for confirming a diagnosis based on less clear manifestations of LB, particularly in regions endemic for the disease, where positive serology may be due to past exposure to *B. burgdorferi* s.l. Direct detection of *Borrelia* in clinical specimens is typically accomplished by culture or PCR [13]. Culture is slow and labor intensive, while PCR has the advantage of sensitivity, simplicity and speed. In addition, real time PCR allows the quantification of organisms present in infected samples, such as animal tissues [14]–[16], ticks [17], human skin [18]–[20] and CSF [21]. In animal studies, it has been shown that the number of *B. burgdorferi* s.s. in tissues is closely correlated with the degree of joint swelling and inflammation [14], [16], [22]. *B. burgdorferi* s.s has been quantified in human skin biopsies [19], however to our knowledge, quantification of *B. afzelii* or *B. garinii* in European human skin biopsies has not yet been reported. We have developed an internally controlled, duplex quantitative real time PCR assay targeting the *Borrelia* single copy 16S rRNA gene and the single copy human RNAseP gene. RNAseP has been extensively used in molecular diagnostic assays to monitor the efficiency of DNA extraction [23], [24]. In addition, by co-amplification of the human RNAseP gene, the bacterial load can be expressed in relation to human genomic DNA, thereby compensating for variations arising from the DNA extraction procedure and/or the size and quality of the biopsy (e.g. variations in tissue composition due to differences in the site from which the biopsy was obtained). We used this assay together with culture, to evaluate skin biopsies from 121 patients presenting with a solitary EM at the University Medical Centre Ljubljana in Slovenia. These data enabled us to compare the ability of both methods to confirm a clinical diagnosis of EM as well as providing information on quantification of *B. burgdorferi* s.l. in skin biopsies. The results of this proof-of-concept study also serve to demonstrate the benefit of this PCR assay to determine the efficacy of a LB vaccine in clinical trials.

## Materials and Methods

### *Borrelia* 16S rRNA Real Time PCR

A TaqMan® assay amplifying and targeting a conserved 139-bp fragment of the gene encoding the *Borrelia* 16S rRNA was designed encompassing the following primers: p16Swt-fwd (5′-GGATATAGTTAGAGATAATTATTCCCCGTTTG-3′) and p16Swt-rev (5′-CATTACATGCTGGTAACAGATAACAAGG-3). The corresponding probe pro16Swt (5′-ACAGGTGCTGCATGGT–3′) was labeled at the 5′-end with the fluorescent reporter 6-carboxy flourescein (6FAM) and at the 3′-end with a Minor-Groove-Binder Non-Fluorescent Quencher (MGBNFQ). The real-time PCR reactions (20 µl final volume) contained 900 nM of each primer, 200 nM probe, 10 µl 2x environmental master mix 2.0 (Applied Biosystems), and the respective indicated amount of template DNA was added in a volume of 5 µl. For assay development, serial dilutions of genomic DNA extracted from the *Borrelia burgdorferi* s.s. strain ZS7 or a synthetic DNA oligonucleotide harboring the 16S target region (Sigma Proligo; 5′-AGGATATAGTTAGAGATAATTA TTCCCCGTTTGGGGTCTATATACAGGTGCTGCATGGTTGTACCCTTGTTATCTGTTACCAGCATGTAATGG-3′) were used. Amplification was carried out on an Applied Biosystems 7900Ht Fast Real-Time PCR System using a cycling protocol of 50°C for 2 minutes, 95°C for 10 minutes followed by 40 cycles of 95°C for 15 seconds and 60°C for 60 seconds. Fluorescent signals were recorded using SDS Software v2.3 (Applied Biosystems) and the automatic baseline option was chosen to have the software calculate the baseline for the detectors, whereas the Cq threshold was set manually.

In order to monitor the recovery of human genomic DNA from clinical skin biopsy samples, the 16S rRNA assay was duplexed with a real-time PCR assay targeting the human single-copy gene encoding the RNA moiety of the human RNAseP enzyme. The RNAseP target region was co-amplified and detected by adding 1 µl of 20x TaqMan® RNAseP control reagent (labeled at the 5′-end with VIC; Applied Biosystems) to the 16S rRNA real-time PCR assay and amplification was carried out as described above. The respective standard curves generated for the singleplex 16S qPCR and duplex 16S/RNAseP assays were used to determine PCR efficiency, slope, R^2^ and Y intercept and this data was used to compare the 16S singleplex and duplex assays. For absolute quantification of the target gene/s in clinical test samples, serial dilutions of a template combining human genomic DNA isolated from blood (Roche) and the synthetic 16S oligonucleotide were used.

All skin biopsy specimens, including the negative controls, were tested in triplicate and every PCR run included a minimum of 3 non template controls (NTC), an extraction control (EXC) and a positive control consisting of *Borrelia*-negative human genomic DNA spiked with 250 genome equivalents of ZS7. For a run to be valid, the positive control had to be 16S/RNAseP positive and NTCs and EXC had to test negative for the presence of 16S. Real-time PCR reactions yielding a Cq value of less than 40 were scored as positive. The threshold for Cq determination was set in the early exponential phase of the amplification curve, using a similar setting for all samples. All samples were tested in triplicate and specimens for which only one 16S rRNA PCR reaction was positive were repeated. The sample was considered *Borrelia*-positive if at least one of the repeat 16S PCR reactions was positive. In addition, at least two out of three RNAseP reactions had to yield a Cq value below 32 and every sample had to test negative for PCR inhibition (SPUD assay; see below).

### SPUD PCR-Inhibition Assay

A TaqMan®-based SPUD assay, modified from that reported previously [25], was performed in parallel to control for the presence of PCR inhibitors (or enhancers) in nucleic acids extracted from the skin biopsy samples. The assay, originally developed as a SYBR Green-based assay, was adapted using a TaqMan® probe for detection (SPUD-SPTM2; 5′-6FAM-CATAGC TTGTGCACACTCA-MGBNFQ-3′) and the primers SPUD-SPTF (5′-ACTTGGCT TTAATGGACCTCCAATT-3′) and SPUD-SPTR 5′-CCGTTTTATGTCTTAC GTGGTGTTC-3′ for amplification. Test samples were spiked with 2,000 copies of the synthetic SPUD oligonucelotide (5′AACTTGGCTTTAATGGACCTCCAATTT TGAGTGTGCACAAGCTATGGAACACCACGTAAGACATAAAACGGCCACATATGGTGCCATGTAAGGATGAATGT-3′; MWG Eurofins) and the results of all test samples were compared to a reference control sample. A difference in Cq of +/−0.5 was considered acceptable evidence for the lack of PCR inhibition/enhancement.

### Nucleic Acid Isolation

Nucleic acids were extracted from skin biopsy specimens and control skin portions using a QIAamp DNA Mini kit (Qiagen) and eluted in 80 µl of AE buffer. Extraction controls were set up in parallel.

DNA from *Borrelia* strains and unrelated microorganisms (see below), was extracted from stock cultures which were thawed and vortexed for 15 seconds. Subsequently, 30 µl of 0.1% Tween 20 in 1x Tris EDTA (pH 7.4) was added to a 20 µl culture aliquot and the samples were heat-denatured for 15 minutes at 95°C. After centrifugation for 5 min at 14,000 rpm and DNA quantification, the indicated amount of total DNA was added in 5 µl for subsequent real time PCR analysis. DNA quantification was performed using a Trinean DropSense96 UV/VIS droplet reader (Trinean nv, Belgium).

### Analytical Specificity

Analytical specificity was determined using 1. A panel of 83 Borrelia burgdorferi sensu lato strains comprising B. burgdorferi s.s., B. afzelii, B. garinii, B. bavariensis sp.nov., B. garinii, B. andersonii, B. bissettii, B. finlandensis sp. nov, B. japonica, B. lusitaniae, B. spielmanii and B. valaisiana. 25 ng of total DNA was used as template for qPCR testing of B.burgdorferi s.l. strains. 2. DNA isolated from 21 unrelated microbial species including Bacillus subtilis subspecies spizizenii, Bacteroides vulgatus, Clostridium sporogenes, Escherichia coli, Pseudomonas aeruginosa, Streptococcus faecalis, Staphylococcus aureus, Staphylococcus epidermidis, Micrococcus luteus, Corynebacterium jeikeium, Bacillus cereus, Brevundimonas vesicularis, Ralstonia pickettii, Sphingobacterium multivorum, Staphylococcus gallinarum, Streptococcus equi zooepidemicus, Corynebacterium renale, Paenibacillus polymyxa, Lactobacillus paracasei spp. Paracasei, Klebsiella oxytoca, Bacillus licheniformis. The amount of template used per reaction was greater than 1.3 µg, thus excluding false negative results. 3. DNA isolated from human skin from 6 healthy control subjects was evaluated. All samples were tested in duplicate.

### Determination of Lower Limit of Detection (95% LOD)

Quantitative sensitivity of the 16S/RNAseP assay was determined using limiting dilutions of genomic DNA extracted from the *B. burgdorferi* s.s. strain ZS7 spiked into a constant amount of human genomic DNA isolated from “*Borrelia*-negative” skin (background matrix). For each dilution (10, 8, 6, 4, 2, 1, 0.1 copies) 12 replicates were analyzed and in total 3 runs were performed on 3 consecutive days (i.e. n = 36 for each dilution). Based on these results the positive cut-off point (LOD) was determined, which is defined as the number of target sequences per volume of sample which can be detected in 95% of test runs (95% detection limit).

### Culture

Culture medium for *B. burgdorferi* s.l. (a modified BSK medium termed BSK-B) was prepared using the following components; 900 ml distilled water, 100 ml CMRL 1066 (10x), 5 g neopeptone, 6 g HEPES, 0.54 g tri- Sodium citrate-Dihydrate, 3 g D(+)-Glucose-Monohydrate, 0.8 g Sodium pyruvate, 0.4 g N-acetyl-D-glucosamine, 0.5 g Yeast extract, 2.2 g Sodium hydrogen carbonate, 200 ml Gelatin solution (3.5%), 81.7 ml Bovine Serum Albumin, (30%), 80 ml rabbit serum (heat inactivated), pH adjusted with 7.6 ml 1 N Sodium hydroxide and final volume adjusted to 1350 ml. Medium was sterilized by filtration and a sterility control was performed for each medium lot. The quality control of each batch of medium was performed to ensure that growth of an inoculum of 10 cells of *B. afzelii* (ACA1), *B. garinii* (VSBM) and *B. burgdorferi* s.s. (ZS7) strains could be supported. Medium was shipped to the clinical site (University Medical Centre Ljubljana) in tubes with 6 ml volumes. Un-used medium was also returned to the test laboratory and quality control was performed to ensure that transport to and from the clinical site did not impair the ability of the medium to support growth of small *Borrelia* inocula. Upon receipt at the test laboratory (Baxter), a further 4 ml modified BSK-B medium was added and the culture tubes were capped tightly and incubated at 33°C ±2°C and 5% ±1% CO_2_ for up to 6 weeks. Samples were inspected by dark field microscopy for potential growth of *B. burgdorferi* s.l. If no spirochetes were detected by dark field microscopy, in 40 viewing fields at a magnification of 400x, after 6 weeks of cultivation, the samples were subcultured (5 ml fresh medium was added to 5 ml of culture) for a further 6 weeks. Samples were deemed culture negative if no spirochetes were detected on sub-culture, thus the maximum time in culture was 12 weeks. Stocks were made from positive cultures. Blood was also cultured from the 121 patients as described previously [26].

### Typing of *Borrelia* Isolates

Isolates were typed according to their *ospA* sequences as outlined previously [27].

### Patients and Controls

This study included 121 untreated patients (aged 16 to 84) from the central part of Slovenia with clinically diagnosed LB, who presented at University Medical Centre Ljubljana between June and December, 2010. All patients had a typical single EM lesion defined according to recently published European criteria [28]. A clinical history was obtained from each patient and relevant clinical details recorded including patient age, patient sex, size of EM lesion, time from appearance of EM to biopsy, date of tick bite, location of EM, presence of central clearing, presence/absence of systemic symptoms (fatigue, headache, myalgia, arthralgia, malaise, fever, dizziness, rigors and nausea), presence/absence of local symptoms (itching, burning and/or pain), history of previous LB and incidence and details of other underlying illness and chronic illness requiring treatment (such as arterial hypertension, hyperlipidemia, osteoporosis, diabetes mellitus, thyroid disease, cardiac rhythm abnormality, psychiatric illness, ischemic heart disease, osteoarthritis, asthma, malignancy or Crohns disease).

Skin taken from control subjects without active LB was tested in order to assess the specificity of the culture and PCR methods. Two pairs of skin biopsies from healthy volunteers were tested blinded by PCR and culture. Skin taken from patients who had undergone cosmetic surgery was also tested by PCR (in total 50 specimens taken from 4 patients).

### Skin Biopsy

Local disinfectant (70% ethanol) and anaesthetic (1% xylocaine, injected) were applied to skin before biopsy. Paired punch skin biopsies (3 mm diameter) were taken from the leading edge of the EM lesion from each patient, one of which was transferred directly to culture tubes containing 6 ml modified BSK-B medium, which were held at room temperature for up to one week prior to shipment to the test laboratory (Baxter). The second biopsy was placed in 70% ethanol for PCR and was stored at +2 to +8°C for up to one week, prior to shipment. Biopsy samples for culture and PCR were transported at weekly intervals with temperature monitoring during shipment, samples for culture were transported at room temperature and samples for PCR transported at +2° to +8°C. Patients, from whom a first biopsy tested culture positive, were invited to attend for re-biopsy 2–3 months after antibiotic treatment, to verify the efficacy of the treatment. Re-biopsies were obtained from 93.8% (61/65) of the patients and these samples also served as post-treatment controls.

### Ethics Statement

The study was approved by the National Medical Ethics Committee of the Republic of Slovenia (No 127/06/10) and conducted according to the principles expressed in the Declaration of Helsinki. Signed written informed consent was obtained from all study participants.

### Statistical Analysis of Clinical Data

Data were summarized as means with standard deviation (SD) or medians with range or interquartile range (IQR) for numerical variables; categorical variables were summarized as frequencies and percentages (%). The number of *Borrelia* targets observed for the different groups was compared using the Mann-Whitney test. Chi-squared test with Yates’ correction was used to compare the proportion of positive biopsies in different groups. A logistic regression model was used to examine the association between PCR biopsy positivity and time from EM to biopsy using restricted cubic splines (RCS) [29] to flexibly model the association, the estimated association was displayed graphically. A similar model was fitted using culture positivity as outcome. Data were analyzed using R statistical language [30].

## Results

### Duplex Quantitative Real Time PCR using Human RNAseP as an Internal Positive Control

A quantitative real-time PCR assay was developed to detect a highly conserved region of the *Borrelia* 16S rRNA gene and the human RNAseP gene simultaneously. The latter target was used for data normalization and as an internal extraction control. A successful multiplexed quantitative real-time PCR assay only yields reliable results if multiplex and singleplex reactions produce similar Cq values for the amplification of a particular target sequence. To assess the quantitative performance of the duplex assay in comparison to the 16S singleplex assay, serial dilutions of the template (either synthetic 16S oligonucleotide or total *B. burgdorferi* s.s. DNA) were amplified. All PCR reactions were carried out in the presence of a constant amount (approx. 250 ng) of human genomic DNA as background matrix. Nine independent PCR runs were set up in each case, with 3 PCR runs performed on 3 consecutive days by 3 different operators.

As shown in Table 1, co-amplification of the human RNAseP did not affect the PCR efficiency, sensitivity or correlation coefficients, since almost identical Cq values were obtained using the 16S singleplex and 16S/RNAseP duplex assay. In addition, when superimposing the recorded fluorescent traces, the exponential phase of the singleplex and duplex real-time PCR reactions were similar (data not shown). Finally, no differences were observed for serial dilutions prepared using either a synthetic 16S oligonucleotide or genomic DNA isolated from the *B. burgdorferi* s.s. strain ZS7 (Table 1), demonstrating that a synthetic template can be used for generating reliable standard curves. Therefore, for quantifying *Borrelia* in clinical specimens, a synthetic 16S oligonucleotide combined with human genomic DNA was used as template for the generation of standard curves.

**Table 1:** Comparison of Cq values (Mean ±1 SD; n = 9) from single and duplex real-time PCR assays obtained with dilution series of *Borrelia* DNA or a synthetic *Borrelia* 16S oligonucleotide in a background matrix of human genomic DNA.

Copies 16S	Synthetic *Borrelia* 16S		Total *Borrelia* DNA	
	16S	16S/RNAseP	16S	16S/RNAseP
10,000	26.37±0.28	26.36±0.20	26.44±0.11	26.46±0.11
1,000	29.78±0.22	29.74±0.19	29.89±0.12	29.87±0.06
100	33.17±0.34	33.32±0.24	33.42±0.32	33.12±0.20
10	36.58±0.46	37.03±1.23	37.00±0.77	36.58±0.55
5	37.30±0.72	37.83±1.16	38.33±1.17	37.74±0.85
2.5	38.84±1.25	38.37±0.84	39.02±1.01	38.45±0.84
1	39.49±0.80	39.10±0.98	39.66±0.58	39.52±0.63
PCR Efficiency	104% ±4%	104% ±6%	98% ±1%	103% ±3%
R^2^	0.99±0.004	0.98±0.01	0.98±0.15	0.99±0.004
Y-Intercept	39.45±0.24	39.48±0.27	40.03±0.19	39.06±1.14
Slope	−3.23±0.08	−3.22±0.13	−3.37±0.12	−3.25±0.06

As a constant amount of human genomic DNA was present in the samples used to generate the standard curves, the impact of increasing amounts of 16S copies on the RNAseP assay could also be assessed. The distribution of the Cq values generated using a singleplex RNAseP (median: 21.90; IQR: 21.82 to 22.23; n = 126) or duplex 16S/RNAseP assay (Median: 21.86; IQR: 21.78 to 22.13; n = 126) was virtually identical. In addition, RNAseP Cq values generated in the presence of either 10,000 copies or 1 copy of 16S template DNA (oligonucleotide or total ZS7 DNA) were also similar (data not shown), indicating that a high copy number of *Borrelia* 16S rRNA does not negatively influence the performance of the RNAseP assay.

Taken together, these data clearly demonstrate that the 16S rRNA and human RNAseP assays can be run in a duplex format without negatively impacting the performance of either real-time PCR assay.

### Assay Specificity

**Figure 1:**
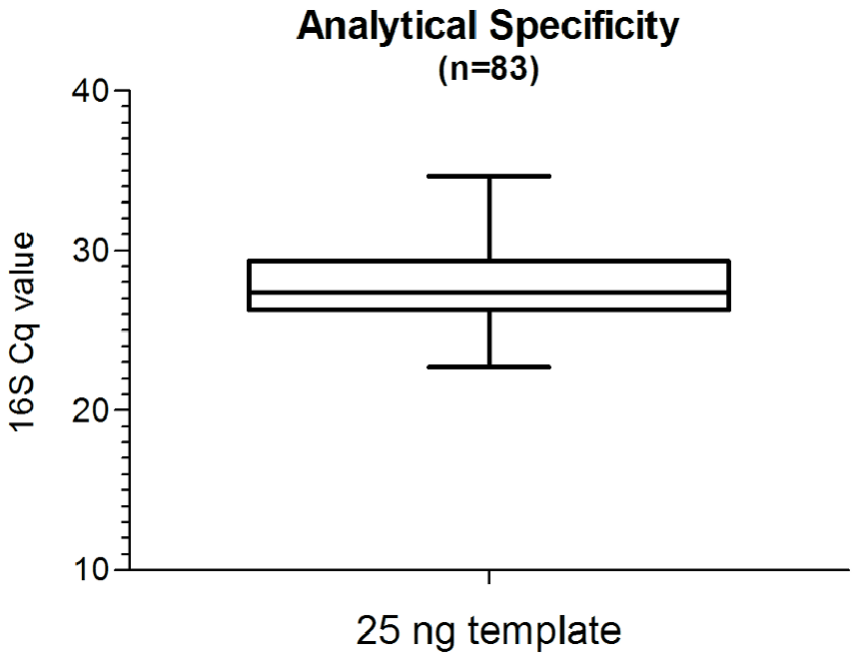
Distribution of Cq values determined for 83 different *B. burgdorferi* s.l. strains. Boxplot analysis of 16S Cq values using the 16S/RNaseP real-time assay probing 25 ng total DNA extracted from a panel of *B. burgdorferi* s.l. including all species known to cause LB. Whiskers indicate the minimal and maximal 16S Cq value determined.

The 16S rRNA gene was selected as the target for PCR, since it is an essential, chromosomally encoded gene and the sequence is highly conserved in *B. burgdorferi* s.l. The assay successfully amplified the conserved 139-bp 16S target using 25 ng of total DNA extracted from a panel of 83 diverse *B. burgdorferi* sensu lato strains selected on the basis of unique *ospA* sequences (data not shown) which included representatives of all major *Borrelia* species causing Lyme disease, notably *B. burgdorferi* s.s. *(n = 15)*, *B. afzelii* (*n = *21), *B. garinii (n = 30)*, *B. bavariensis sp.nov. (n = 4)*, *B. andersonii* (*n = *2), *B. bissettii* (*n = *1), *B. finlandensis sp.nov. (n = 2)*, *B. japonica* (*n = *2), *B. lusitaniae* (*n = *1), *B. spielmanii (n = 3)* and *B. valaisiana* (*n = *2). As shown in Figure 1, the distribution of the determined Cq values was comparable, with a median Cq of 27.4 and an IQR from 26.28 to 29.36. Importantly, the assay was highly specific as it did not amplify DNA from 21 unrelated microorganisms, nor was amplification evident from *Borrelia*-negative human DNA (data not shown).

### Quantitative Sensitivity and Determination of Detection Limit using the Duplex Assay

Quantitative sensitivity of the duplex PCR assay was evaluated using a background matrix of human genomic DNA spiked with limiting dilutions of *B. burgdorferi* sensu stricto DNA (ZS7). In this case, 12 replicates of each dilution (10, 8, 6, 4, 2, 1 and 0.1 *Borrelia* genome equivalents) were tested in 3 runs performed on 3 consecutive days (i.e. n = 36 for each dilution, Table 2). The limit of detection was determined as 6 target copies per PCR reaction (20 µl) and the PCR reaction was positive on 97.2% occasions (Table 2).

**Table 2:** Limit of detection of the 16S rRNA qRT-PCR assay in the presence of human genomic DNA.

	Copies ZS7 genome equivalents						
No. target copies*	10	8	6	4	2	1	0.1
Percent positive (No) **	100% (35/35)	97.2% (35/36)	97.2% (35/36)	86.1% (31/36)	58.3% (21/36)	33.3% (12/36)	5.6% (2/36)

* Dilution series of *Borrelia* DNA from strain ZS7 in a matrix of human genomic DNA.

** Positive samples have Cq <40. Probability of detecting various copy numbers of ZS7 genomes demonstrated. The number of positive samples per tested dilution (typically 36) is indicated in brackets.

### Clinical Data

Paired skin biopsies were taken from 121 untreated adult patients with solitary EM lesions between June and December 2010. Their median age was 54 years (IQR: 43 to 61), and seventy (57.9%) were female. Fifty three (44%) patients had EM skin lesion with central clearing, 29 (24%) reported systemic symptoms in addition to EM, and 48 (40%) complained of symptoms at the site of the skin lesion (mild itching, burning and/or pain). Forty eight (39.7%) patients had an underlying chronic illness requiring treatment, while 7 (5.8%) had cancer. Twenty nine (24%) patients presenting with EM lesion had a history of previous LB, 4 (13.8%) of whom were diagnosed with extracutaneous manifestations of LB with/without EM at first presentation.

### Evaluation of Clinical Samples and Controls by PCR and Culture

The skin biopsy samples collected were evaluated by real time PCR and culture (Table 3). In total, 3 cultures became contaminated and hence could not be evaluated. PCR identified more positive biopsy samples, with 94/121 positive (77.7%) compared to 65/118 (55.1%) biopsies positive by culture. None of the samples showed signs of PCR inhibition. Importantly, all specimens that were positive by culture were also positive by PCR. DNA isolated from biopsy samples was tested in triplicate by real-time PCR and for 8 of the 121 samples (6.6%), seven of which were culture negative, only one of three technical replicates tested *Borrelia* positive. However, as repeat testing yielded a minimum of one positive result, the samples were considered positive for *Borrelia* infection, albeit with a low copy number (0.2 to 7 *Borrelia* 16S copies per 10,000 human genome equivalents). RNAseP values for these samples indicated successful DNA extraction and there were no signs of PCR inhibition. Control biopsies which were obtained from 2 healthy volunteers and from 4 subjects who had undergone plastic surgery, were correctly identified as PCR negative, which was confirmed by repeat testing. Finally, all re-biopsies taken from the patients after antibiotic treatment (n = 61) were culture negative, although one re-biopsy tested PCR positive.

**Table 3:** Comparison of PCR and culture for the detection of *B. burgdorferi* s.l. in EM lesions and comparison of median copy number in culture positive and culture negative biopsies.

Detection by :		Number (%)	Median copies (IQR)
PCR	Culture		Per 10,000 human genome equivalents
+	+	65 (55.1%)^a^	22 (6–53)*
+	−	27 (22.9%)	6 (2–31.5)
−	−	26 (22.0%)	0
−	+	0	0

* No of spirochetes is significantly higher (P<0.001, Mann-Whitney test all patients, P = 0.04 for PCR+ patients) in culture+versus culture – biopsies.

a 3 cultures became contaminated, (2 PCR positive and 1 PCR negative), hence 118 samples could be evaluated by culture.

Biopsy samples were collected over a one week period (stored at room temperature for culture and at 4°C for PCR) and shipped to the test lab weekly. The effect of the length of the holding time, between taking biopsies to their incubation at 33°C, on subsequent culture isolation rates was investigated in the subset of patients who had PCR confirmed EM (n = 92, 2 PCR positive samples which were culture contaminated were excluded from analysis).

For PCR positive samples processed rapidly (incubation at 33°C ≤2 days after biopsy) 65% (17/26) of the samples were culture positive, while for samples processed less rapidly (incubation at 33°C >2, ≤8 days after biopsy) 73% (48/66) were culture positive; the difference was not statistically significant (*P* = 0.66, Chi-squared test with Yates’ correction). Similar results were obtained for the complete data set [46% (17/37)] and for culture positives [59% (48/81)], for rapidly and less rapidly processed samples, respectively, (*P* = 0.25, Chi-squared test with Yates’ correction).

There was also no difference in how rapidly the cultures became positive, 11.7% (2/17) of those processed more rapidly (incubation at 33°C ≤2 days after biopsy) were culture positive in less than 6 weeks, versus 12.5% (6/48) of those processed less rapidly (incubation at 33°C >2, ≤8 days after biopsy, P>0.99). Thus, the length of holding time at room temperature prior to incubation (≤8 days) appeared to have no detrimental effect on the isolation rate of *Borrelia* in culture.

### Quantitative PCR Results in Culture Positive and Culture Negative Specimens

**Figure 2:**
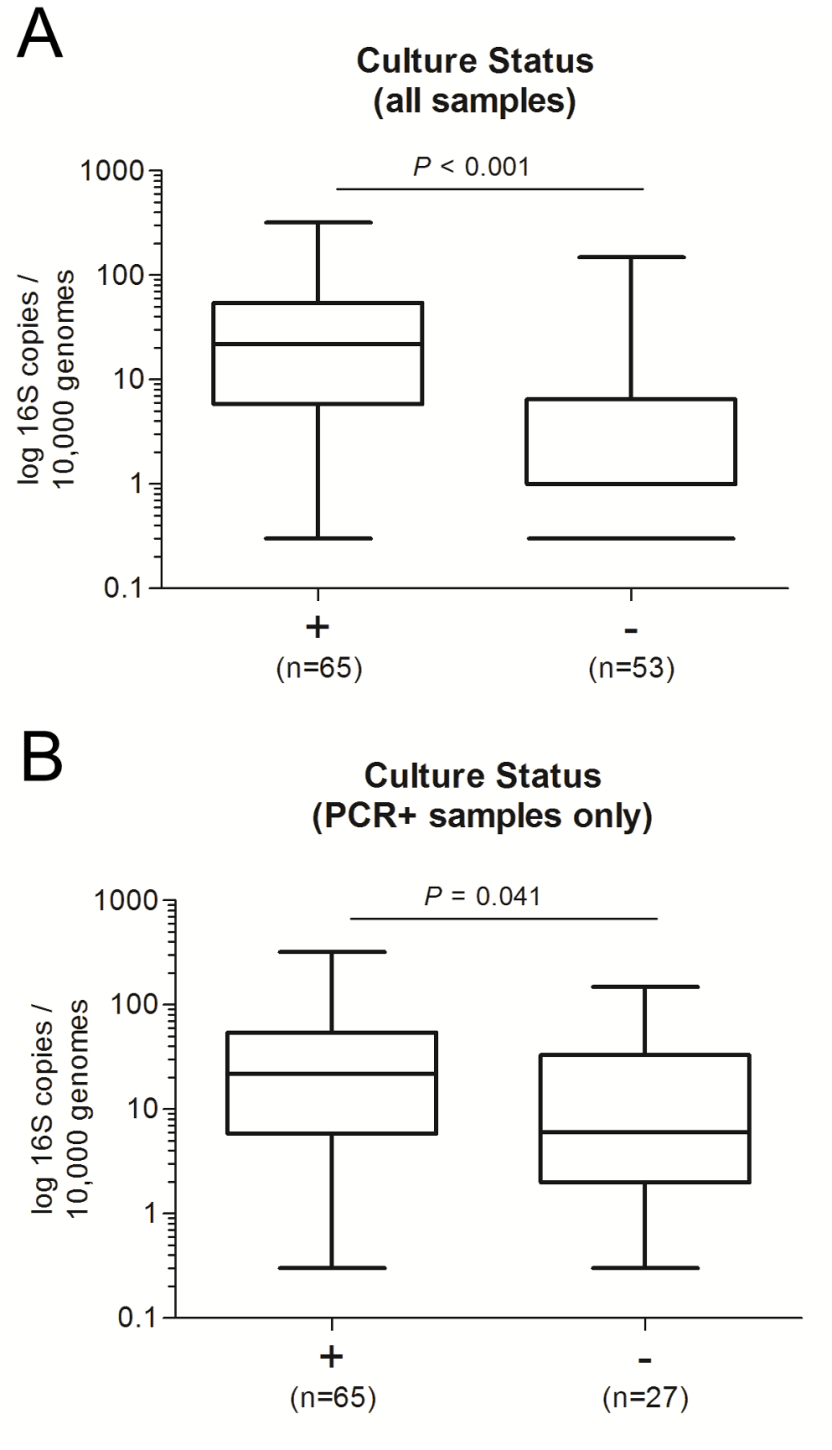
16S copy number of *Borrelia* positive and negative cultures. Boxplot showing the 16S copies (log_10_) detected in skin biopsy specimens for which the paired biopsies were either positive or negative for *Borrelia* in culture. (**A)** Analysis for all samples and (**B)** only samples which were positive by PCR. Whiskers indicate the minimal and maximal copy number determined. Data were analyzed using the Mann–Whitney test.

The number of *Borrelia* 16S rRNA targets detected by qPCR for PCR positive patients varied considerably, ranging from 0.2 to 321 *Borrelia* 16S rRNA target copies per 10,000 human genome equivalents with a median of 15 (IQR: 5 to 39.75). The median number of *Borrelia* 16S rRNA targets detected in culture-positive specimens was significantly higher than in culture-negative specimens (Figure 2; P = <0.001 considering all patients and P = 0.04 for PCR positive patients, Mann-Whitney test, Table 3). RNAseP was detected for all samples (n = 121) and the copy number per reaction ranged from 5,364 to 131,027, with a median of 29,640 (IQR: 21,080 to 44,220). For *Borrelia* PCR positive biopsies (n = 94), the RNAseP per PCR reaction ranged from 5,364 to 131,027 (median 30,849, IQR: 22,170 to 38,800). While for *Borrelia* PCR negative biopsies (n = 27), the RNAseP value per PCR reaction ranged from 6,471 to 62,531 (median 26,122, IQR: 17,900 to 39,690).

### Typing of *Borrelia* Strains Isolated in Culture

*Borrelia* isolated in culture were typed (62/65 cultures) by sequencing a large fragment of the *ospA* gene as outlined previously [27]. Sixty of the isolates (96.8%) had an OspA sequence which was identical (82.3%, 51/62) or highly similar (99% OspA sequence identity) to the OspA sequence for *B. afzelii* strain PKo (GenBank Acc. No. S48322 [31]). The remaining two isolates had an OspA sequence which was identical to the OspA sequence for *B. garinii* strain Tlsl (GenBank Acc. No. X85440 [32]).

### Relationship between Clinical Picture and PCR Quantification

**Figure 3:**
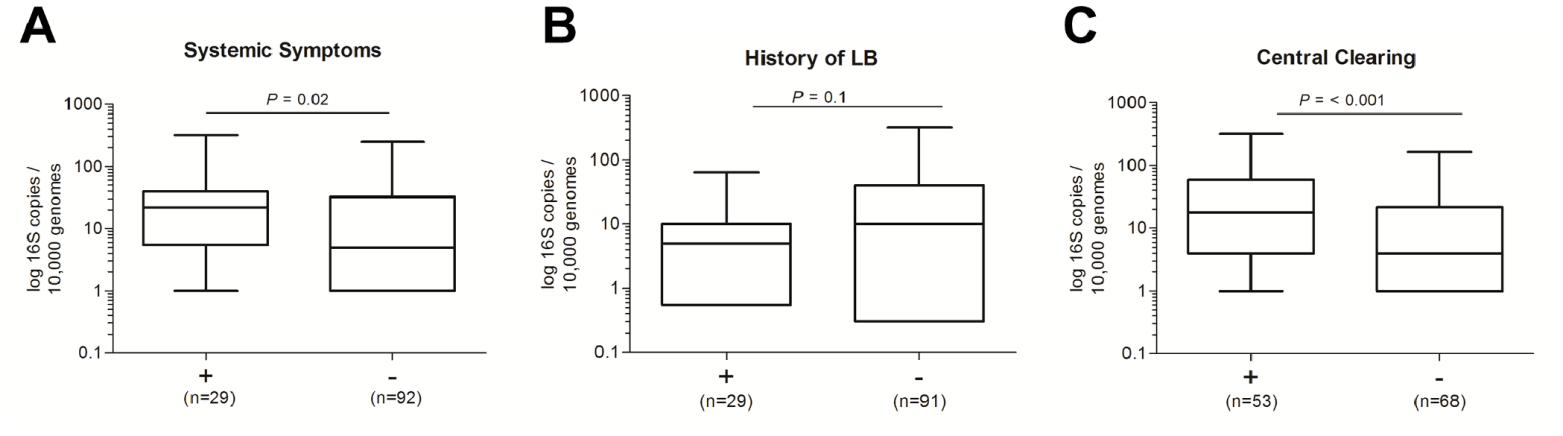
Association of *Borrelia* 16S rRNA targets per 10,000 genome equivalents and clinical symptoms. (**A**) Boxplot showing log_10_ 16S copies determined for biopsy specimens from patients presenting with (+) or without (−) systemic symptoms. (**B**) Distribution of *Borrelia* 16S copies quantified from specimens taken from patients reporting a previous diagnosis of LD. (**C**) 16S copies quantified for biopsies taken from EM lesions with or without central clearing. Whiskers indicate the minimal and maximal copy number determined. Data were analyzed using the Mann–Whitney test.

Systemic symptoms such as fatigue, headache, myalgia, arthralgia, malaise, rigors, dizziness, nausea and fever (Table 4) were reported by 29 patients (24%). The median number of *Borrelia* per human 10,000 genome equivalents was significantly higher (P = 0.02) in patients with systemic symptoms than without (Figure 3A and Table 4). This association was not statistically significant for any individual systemic symptom (data not shown). Of the 29 patients with systemic symptoms 26 (89.7%) were PCR positive and 17 (58.6%) were culture positive for *B. afzelii*. Two of these patients also had a positive blood culture (*B. afzelii*), only one other patient, who did not report systemic symptoms, had a *B. afzelii* positive blood culture. All three patients from whom blood cultures were positive also had positive skin biopsy cultures.

**Table 4:** Summary of clinical characteristics for 121 patients with EM and association of the significance of spirochete number with clinical characteristic.

	Present		Absent		
Characteristic	Numberpatients (%)	Median copy number per 10,000 human genomes (IQR)	Numberpatients (%)	Median copy number per 10,000 human genomes (IQR)	*P* a
Systemic symptoms^b^	29 (24%)	22 (6 to 39)	92 (76%)	5 (0 to 31.5)	0.02
Symptoms at EM site^c^	48 (40%)	12.5 (0 to 36.25)	73 (60%)	6 (1 to 25)	0.72
History of LB^d^	29 (24%)	5 (0.8 to 9)	91 (76%)	10 (0.65 to 39.5)	0.10
Underlying illness^e^	55 (45%)	5 (1 to 35)	66 (55%)	7.5 (0.3 to 33)	0.92
Immunocompromised^f^	6 (5%)	5 (0.25 to 33.75)	115 (95%)	6 (0.9 to 33.5)	0.81
Central clearing	53 (44%)	18 (4 to 56)	68 (56%)	4 (0 to 21.25)	<0.001

a As determined by Mann-Whitney test.

b Systemic symptoms comprised fatigue, headache, myalgia, arthralgia, malaise, rigors, dizziness, nausea and fever.

c Symptoms at site comprised itching, burning and pain.

d History of extracutaneous LB and/or EM, previously diagnosed by a physician; data not available for 1 patient (i.e.) n = 120.

e Chronic underlying illness; arterial hypertension, hyperlipidemia, osteoporosis, diabetes mellitus, thyroid disease, cardiac rhythm abnormality, psychiatric illness, ischemic heart disease, osteoarthritis or asthma.

f Patients had malignancy and were treated with chemo−/radiotherapy within the last year.

Local symptoms at the biopsy site were reported by 48 patients (40%) and comprised itching, burning and pain. Thirty five of the patients with local symptoms (72.9%) were PCR positive and 23 (47.9%) were culture positive, but there was no significant association between these symptoms and the number of spirochaetes (*P* = 0.72, Table 4).

Of the twenty nine patients who had a previous history of LB, 23 (79.3%) and 16 (55.1%) were PCR and culture positive respectively. The median *Borrelia* 16S rRNA target copy number was 2-fold lower in biopsies from patients who reported a previous history of LB (*P* = 0.10, Figure 3B and Table 4).

Fifty five of the biopsies (45%) were from patients who had an underlying illness, but there was no significant association between underlying illness and the number of 16S rRNA *Borrelia* targets or the probability of a positive result by PCR or culture (*P* = 0.92). Six of the patients were immunocompromised with reported malignancy (patients diagnosed with malignancy with current chemo- or/and radiotherapy, or chemo−/radiotherapy within the last year). Copy number in these patients was comparable to copy number in non-immunocompromised patients.

Central clearing of EM lesions was observed significantly more often among patients with positive PCR (50% vs 22%, *P* = 0.02), also the number of spirochetes was significantly higher among patients with central clearing (*P*<0.001, Figure 3C and Table 4). There was no association between central clearing of EM lesions and systemic symptoms (*P* = 0.93).

**Figure 4:**
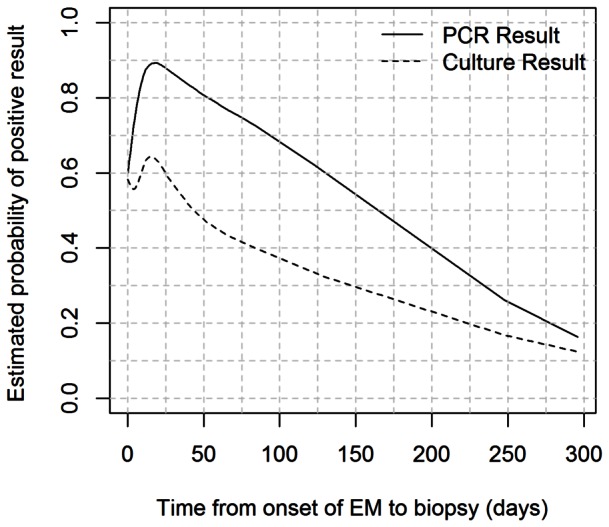
Estimated probability of positive detection of *B. burgdorferi* s.l. by culture and PCR as a function of time from onset of EM to biopsy.

Time from the onset of EM (as appreciated by patients) to biopsy ranged from 0 to 296 days (median 10.5 days). The association between time from EM to biopsy and PCR and culture positivity was also investigated (Figure 4). The probability of a positive result markedly increased with time from biopsy for the biopsies taken within about 20 days (*P* = 0.07), while it decreased afterwards. The association between culture positivity and time from EM to biopsy was not statistically significant (*P* = 0.45), but the shape of the estimated association closely mirrored the results obtained for PCR positivity (Figure 4).

## Discussion

Conventional diagnosis of LB is often based solely on the evaluation of an EM lesion. However, due to the high degree of variability of skin lesions and the subjective nature of the interpretation, misdiagnosis is possible. Isolating *Borrelia* from skin biopsy specimens is considered the gold-standard, however it is time consuming and does not contribute to early diagnosis [13]. Quantitative real-time PCR for direct and rapid molecular detection and quantification of pathogens has become a widely used technology for clinical applications and can be valuable for confirming a diagnosis based on less clear manifestations of LB or for investigating controversial disease syndromes attributed to infection with *B. burgdorferi* s.l. In addition, PCR testing can complement serology testing in endemic areas for LB, where positive serology may be due to past exposure to *B. burgdorferi* s.l.

Numerous real time PCR assays for the detection of *B. burgdorferi* s.l. have been reported previously, however few have included an internal control [21], [22] nor has a quantitative PCR study of *Borrelia* in European clinical skin biopsy samples been published to date.

We have developed a *Borrelia* species-specific TaqMan® probe–based qPCR assay targeting the 16S rRNA gene of all *B. burgdorferi* s.l. strains known to cause LB. The inclusion of an assay simultaneously targeting the human RNAseP gene as an internal control enabled sample-to-sample normalization of the bacterial burden (i.e. copies of *Borrelia* 16S rRNA per 10,000 human genome equivalents) and allowed successful DNA extraction to be monitored. The assay described is highly sensitive, detecting 6 copies of 16S rRNA gene per PCR reaction, which is similar to values reported for *B. burgdorferi* s.l. from previous studies [7], [21]. Furthermore, the assay is highly specific, as it failed to detect the 16S rRNA gene of 21 unrelated microbial species or any bacterial DNA that may have been co-extracted from skin commensals present in control skin biopsies. Based on these observations, we are confident that the assay can detect all known pathogenic *B. burgdorferi* s.l. in clinical skin biopsy samples.

To evaluate the diagnostic performance of the real-time qPCR assay in comparison to culture for detecting *B. burgdorferi* s.l. from skin biopsies, we undertook a study of paired 3 mm skin biopsy samples taken from 121 patients with typical solitary EM [28].

PCR testing identified more positive samples than culture (77.7% PCR positive versus 55.1% culture positive) and correctly identified all specimens scored as positive by culture. Importantly, all control samples tested negative for the presence of *B. burgdorferi* s.l. nucleic acids. In comparison, PCR assays for detection of *Borrelia* from skin biopsies described previously have reported sensitivity ranging from 25% [33] to 88% [34]. Finally, 96.8% of the isolates were typed as *B. afzelii*, which is consistent with previous studies conducted in the central part of Slovenia where *B. afzelii* accounted for approximately 86% of the strains isolated [35]–[37].

The median number of 16S rRNA targets per 10,000 human genome equivalents was significantly higher in culture positive biopsies than in culture negative biopsies, which is consistent with findings reported from quantitative PCR studies of *B. burgdorferi* s.s. in skin biopsies [19]. However, although we observed a clear relationship between a high *Borrelia* DNA load and culture positivity, individual samples failed to grow in culture despite having a high *Borrelia* DNA load. Possibly the distribution of *Borrelia* may have differed between the 2 separate biopsy samples used for culture and PCR [33] or particular strains may grow less well in culture medium or may not have been viable in the retrieved biopsies.

There was a significant association between the estimated probability of PCR and culture positivity and time from the onset of EM to biopsy. The probability of a positive result increased within the first 20 days after the appearance of EM and decreased afterwards, possibly reflecting dissemination of spirochetes from the EM lesion, and/or a decrease in spirochete numbers due to the host immune response. Our results are in line with a previous study reporting an inverse relationship between the recovery of *B. burgdorferi* s.s. from a skin specimen and EM duration [38]. Furthermore, significantly fewer spirochetes have been reported in older EM lesions [19]. Therefore, the possibility of confirming a clinical diagnosis of EM by either PCR or culture is potentially lower for long standing lesions.

Biopsies were also taken after completion of antibiotic treatment (post-treatment biopsies) in subjects from whom the first biopsy was culture positive. All post-treatment biopsies were culture negative and, all but one, were PCR negative. The single PCR positive (and culture negative) post-treatment biopsy was taken from a subject who responded well and whose EM disappeared after initial treatment, with no further antibiotic intervention deemed necessary. These results suggest that the infection had recently resolved and that no viable *Borrelia* were present, highlighting a potential limitation of PCR-based assays in terms of assessing on-going infection (especially after treatment), where the detection of DNA may indicate a recent infection and does not necessarily mean that live *Borrelia* are present.

A clear relationship between clinical symptoms and *Borrelia* burden was evident from this study. The median number of 16S targets detected per 10,000 human genomes was significantly higher among patients with systemic symptoms than patients who did not report systemic symptoms. A positive correlation between clinical symptoms and spirochete burden has been reported in the mouse model [14], [16], [22], however this is the first study demonstrating an association between spirochete burden and clinical disease symptoms in humans. The median number of 16S targets was not different in patients with another underlying disease. Also no association between local symptoms at the site of EM and 16S rRNA target copy number was found, although host specific factors may likely contribute to local symptoms.

EM is described as an expanding erythematous skin lesion with or without central clearing [28]. In our study which comprised predominantly *B. afzelii* isolates, central clearing of EM lesions was observed more often among patients with positive PCR and the number of spirochetes was significantly higher among patients with central clearing. These findings may pertain only to EM caused by *B. afzelii* and may not be valid for EM lesions caused by other *Borrelia* species such as *B. burgdorferi* s.s. and *B. garinii*, since different *Borrelia* species cause distinct clinical presentations [39], [40]. Possibly the strains isolated from cases of EM with central clearing evoked a less intense host response, which is less likely to result in spirochaetal killing.

To our knowledge, this is the first quantitative PCR study to have evaluated patients with a history of LB. The median number of 16S rRNA targets was 2 fold lower in patients with a history of LB. Re-infection in patients with LB has been reported in Europe and in the US [41], [42]. In a study of patients with EM, [43] *B. burgdorferi* s.s. was significantly less likely to be recovered from blood samples of patients with a prior history of LB, implying that partial immunity may protect against disseminated infection. Moreover, studies in dogs have shown that the number of *B. burgdorferi* s.s organisms detected in skin biopsies was inversely correlated with the antibody levels measured by ELISA [16]. Knowledge of the immune status of the individuals with a reported history of LB at the time of re-infection was not determined during this study, however it is possible that prior immunity may have limited the bacterial burden during a subsequent infection.

In conclusion, we have developed an internally controlled, sensitive duplex 16S rRNA/RNaseP qPCR assay specific for all *B. burgdorferi* s.l. strains known to cause LB. The results presented demonstrate that the assay is a sensitive, rapid and reliable approach for detection of *Borrelia* in skin biopsy samples retrieved from EM lesions and support its use for laboratory confirmation of clinically diagnosed LB, e.g. to assess the efficacy of a LB vaccine during clinical trials. Furthermore, this is the first quantitative PCR study of human skin biopsies predominantly infected with *B. afzelii* and the first study to report a clear relationship between clinical systemic symptoms and *Borrelia* target copy number.
